# Multi-Graph Multi-Label Learning Based on Entropy

**DOI:** 10.3390/e20040245

**Published:** 2018-04-02

**Authors:** Zixuan Zhu, Yuhai Zhao

**Affiliations:** College of Computer Science and Engineering, Northeastern University, Shenyang 110819, China

**Keywords:** multi-graph multi-label, entropy, informative subgraphs, extreme learning machine

## Abstract

Recently, *Multi-Graph Learning* was proposed as the extension of *Multi-Instance Learning* and has achieved some successes. However, to the best of our knowledge, currently, there is no study working on *Multi-Graph Multi-Label Learning*, where each object is represented as a bag containing a number of graphs and each bag is marked with multiple class labels. It is an interesting problem existing in many applications, such as image classification, medicinal analysis and so on. In this paper, we propose an innovate algorithm to address the problem. Firstly, it uses more precise structures, multiple *Graphs*, instead of *Instances* to represent an image so that the classification accuracy could be improved. Then, it uses multiple labels as the output to eliminate the semantic ambiguity of the image. Furthermore, it calculates the entropy to mine the informative subgraphs instead of just mining the frequent subgraphs, which enables selecting the more accurate features for the classification. Lastly, since the current algorithms cannot directly deal with graph-structures, we degenerate the *Multi-Graph Multi-Label Learning* into the *Multi-Instance Multi-Label Learning* in order to solve it by *MIML-ELM (Improving Multi-Instance Multi-Label Learning by Extreme Learning Machine)*. The performance study shows that our algorithm outperforms the competitors in terms of both effectiveness and efficiency.

## 1. Introduction

Due to the advance of smart phones, nowadays people upload a great number of photos to the Internet. Updating photos has become easier, but searching them becomes more difficult. Though the technology of searching images by images has appeared, most people rely on the traditional way to searching an image, which is searching images by typing keywords. For that, we need to add labels for each image, but it cannot be accomplished by human beings due to the great number of unlabeled images. Thus, it is important to use Machine Learning to automatically classify images [1,2] and add correct labels for them.

**Figure 1:**
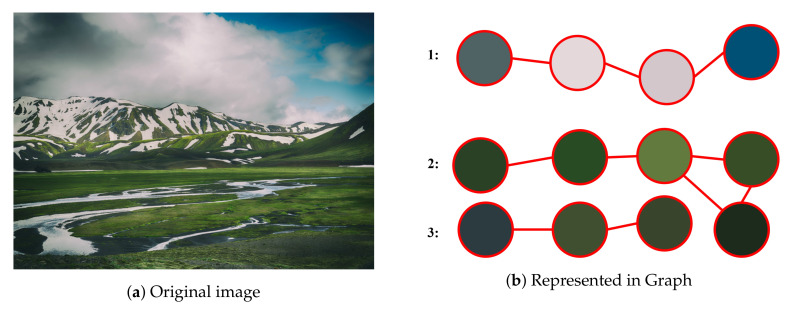
An example figure with structure Graph. (**a**) Original image; (**b**) Represented in Graph.

*Multi-Instance Learning* is extensively studied in image classification [3]. It uses a kind of data structure called *Feature Vector* to represent a real image [4], but it is a little bit imprecise, since vectors can only show the pixels of images without the adjacency relations between pixels. Thus, it is natural to consider that *Graph* may be a better data structure to represent an image [5] because a *Graph* consists of edges and nodes. Nodes can indicate the texture or color of pixels in an image and the edges can indicate the adjacency relations of nodes. Like the following image in Figure 1a, a *Feature Vector* can only tell you there are white, blue and green pixels in this image, whereas, after segmenting the image into three *Graphs* [6] in Figure 1b, *Graphs* can show the adjacency relations between each pixel. (In the real application, the image will be segmented into more graphs with more nodes.) The latter one represents more real details of an image, which will be more beneficial for the accuracy in the learning part.

Recently, a graph-structure algorithm, namely *gMGFL* [7,8,9,10], was proposed by Jia Wu et al. Briefly, it works in the following steps. Firstly, there are many different images in the training dataset. *gMGFL* segments each image into multiple graphs, all which are packed into a graph bag. Secondly, the label is only visible for the graph bag and each graph bag will only be marked with one label. For a specific subject, if an image contains it, the label of the graph bag for this image will be positive; on the contrary, it will be negative. Thirdly, to build an appropriate classifier, it needs to mine some informative subgraphs, which can stand for the traits of the subject in the images (or the traits of the subject not in the images), and use these subgraphs as the features for classifying. It is brilliant, but it still has some drawbacks. Two major problems of *gMGFL* are listed as below.

**Figure 2:**
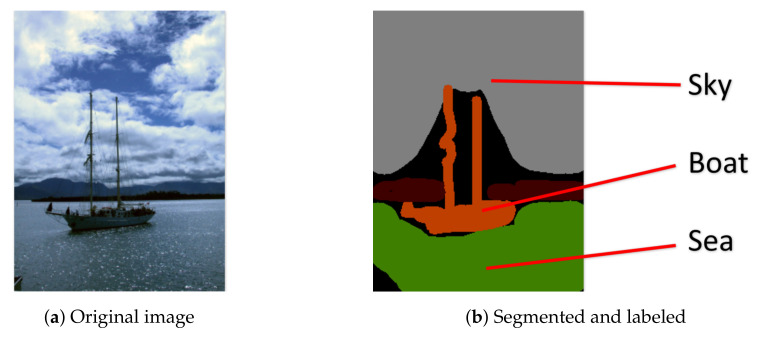
An example figure with Multi-Label. (**a**) Original image; (**b**) Segmented and labeled.

Firstly, in the algorithm *gMGFL*, each image will only be marked with one label, so it can only deal with one subject. Nevertheless, in the real life, it is impossible that an image just contains one subject. It often includes multiple semantic information [11]. For example, in Figure 2, the image contains three different subjects: sea, boat and sky, so it should be marked with three kinds of positive labels (and maybe also marked with some negative labels, like lion or apple). It cannot mark the image with only one label, like *sea*. Otherwise, it will cause some problems: if the user types a searching keyword *boat* or *sky*, this image will not be shown in the result. Unfortunately, *gMGFL* can only deal with a one-label problem.

**Figure 3:**
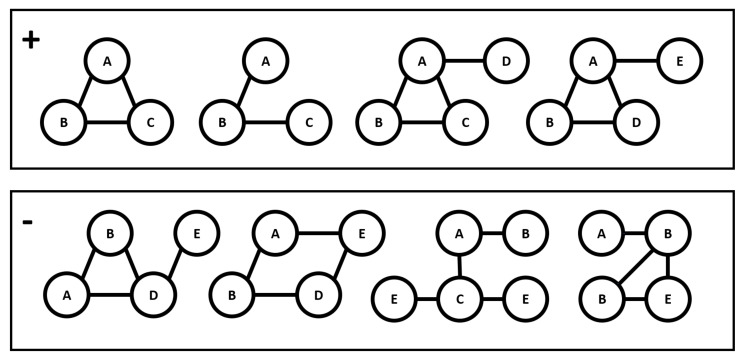
A graph dataset with class label.

Secondly, in the part of mining informative subgraphs, *gMGFL* considers that if a subgraph is informative, it should be frequent in the dataset. Therefore, *gMGFL* mines the frequent subgraphs [12] and uses them as the features for classifying, but this is not always accurate. In Figure 3, there are eight graphs and two different classes. The above four are marked with positive labels and belong to the positive class; the four below are marked with negative labels and belong to the negative class. If we only consider the frequent-subgraph mining, the subgraph A−B has the frequency of eight because it appears eight times in all graphs. Nevertheless, if we regard it as an informative subgraph (a.k.a. classifying feature), it cannot distinguish the positive class from the negative class, since it is a common feature between two classes. Not only do all positive graphs contain the subgraph A−B, but also all negative ones contain it as well. Thus, the subgraph A−B is not appropriate to be an informative feature, but, due to its high frequency, *gMGFL* considers that it is, which will cause imprecise results.

To solve these problems, in this paper, we proposed an advanced graph-structure algorithm named *Multi-Graph Multi-Label Learning*. This algorithm may also be utilized in calculating the similarity of biological sequences, predicting the function of chemical compounds, analyzing structured texts such as HTML (Hypertext Markup Language) and XML (Extensible Markup Language), etc. The following are our major contributions:

1. Our algorithm is based on a multi-graph and it can also solve multi-label (i.e., multiple subjects) problems, which means it can deal with multiple semantic information. To the best of our knowledge, we are the first one to propose such an algorithm.
2. We introduce a novel subgraph-mining technique called *Entropy-Based Subgraph Mining*. It calculates the information entropy for each graph [13] and uses this criterion to mine the informative subgraphs, which is more suitable than the one based on frequent-subgraph.
3. In the part of building the classifier, we utilize the algorithm *MIML-ELM* (we will discuss it briefly in Section 2.3). It uses the *Extreme Learning Machine* rather than *Support Vector Machine* to build an image classifier, which is more efficient.

The rest part of this paper is organized as the following. Related works are introduced in Section 2. The algorithm description of *Multi-Graph Multi-Label Learning* is presented in Section 3. The results of our experiments are provided in Section 4. Our conclusions are in Section 5.

## 2. Related Work

The research in this paper is related to some previous works of graph-structure classification, *Multi-Instance Multi-Label Learning* and *MIML-ELM*. We will briefly review them respectively in Section 2.1, Section 2.2 and Section 2.3.

### 2.1. Graph-Structure Classification

There are two kinds of algorithms about graph-structure classification: one of them is based on global distance and the other one is based on subgraph feature, and it has been proved that the subgraph-feature approach is better [14]. It converts a set of subgraphs into feature vectors so that most of the current algorithms can be utilized in the graph classification problem. Almost all these kinds of algorithms (such as *AGM* [15], *Gaston* [16], *gSpan* [17,18], *gHSIC* [19,20]) extract subgraph features by using frequent substructure pattern mining, and the most widespread mining algorithm among them is *gSpan*.

### 2.2. Multi-Instance Multi-Label Learning

*Multi-Instance Multi-Label Learning* is a supervised learning algorithm, which represents real-world objects with bags of instances and labels. The most widespread algorithm is *MIML-SVM* [21]. It degenerates *Multi-Instance Multi-Label* to *Single-Instance Multi-Label* by clustering multiple labels to binary classification tasks with *Support Vector Machine*. Zhou et al. proposed *MIML-SVM* in [22] and recently Li et al. improved it in [23].

### 2.3. MIML-ELM

The full name of *MIML-ELM* is *Improving Multi-Instance Multi-Label Learning by Extreme Learning Machine* [24]. *Extreme Learning Machine* (*ELM*) is one of the models in Neural Networks and is extensively utilized in *Single Hidden-layer Feed-forward Network*. Recently, Li et al. proposed an efficient and effective algorithm named *MIML-ELM*, which utilized the *ELM* in solving the *Multi-Instance Multi-Label* problem. Firstly, this algorithm is more effective in the process of degeneration from *Multi-Instance Multi-Label* to *Single-Instance Multi-Label*, since it provides a theoretical guarantee to automatically determine the number of clusters. Secondly, this algorithm is more efficient, since it uses *ELM* instead of *Support Vector Machine* to improve the two-phase framework.

## 3. The MGML Algorithm

This section is about the algorithm description of *Multi-Graph Multi-Label Learning* (*MGML*). Firstly, we will introduce some relative concepts in Section 3.1. Then, we discuss our proposed approach in Section 3.2, Section 3.3 and Section 3.4. Lastly, an illustrative example of *MGML* is given in Section 3.5.

### 3.1. Problem Definition

The following are some basic definitions about our algorithm.

**Definition** **1.** ***(Graph Bag):*** *G is a graph denoted as G=(N,E,L,l). N is a set of nodes; E is a set of edges and E⊆N×N; L is a set of labels for nodes and edges; l is the function mapping labels to nodes and edges and l:N∪E→L. A graph bag $Bag=\{G_{1},\cdots ,G_{j},\cdots ,G_{n}\}$ contains n graphs, where $G_{j}$ denotes the j-th graph in the bag.*

**Definition** **2.** ***(Subgraph):*** *subgraph Given G=(N,E,L,l) and $SubG_{k}=\left(N^{\prime },E^{\prime },L^{\prime },l^{\prime }\right)$, we say that $SubG_{k}$ is a subgraph of G, if and only if there exists a function $\psi :N^{\prime }\to N$ s.t. (1) $\forall n\in N^{\prime },l^{\prime }\left(n\right)=l\left(\psi \left(n\right)\right)$; (2) $\forall \left(n_{1},n_{2}\right)\in E^{\prime },\left(\psi \left(n_{1}\right),\psi \left(n_{2}\right)\right)\in E$, and $l^{\prime }\left(n_{1},n_{2}\right)=l\left(\psi \left(n_{1}\right),\psi \left(n_{2}\right)\right)$. In addition, we can say that G is a super-graph of $SubG_{k}$.*

**Definition** **3.** ***(Subgraph Feature Representation for Graph):*** *Let $Set_{SubG}=\left\{SubG_{1},\cdots ,SubG_{s}\right\}$ be a set of subgraphs mined from a graph set $Set_{G}=\left\{G_{1},\cdots ,G_{t}\right\}$. For each graph $G_{i}\left(i\in \left[1,t\right]\right)$ in $Set_{G}$, we use a feature vector $X_{i}^{G}={\left[{\left(X_{i}^{SubG_{1}}\right)}^{G},\cdots ,{\left(X_{i}^{SubG_{k}}\right)}^{G},\cdots ,{\left(X_{i}^{SubG_{s}}\right)}^{G}\right]}^{T}$ to represent it. ${\left(X_{i}^{SubG_{k}}\right)}^{G}=1$ if and only if $SubG_{k}$ is a subgraph of $G_{i}$. Otherwise, ${\left(X_{i}^{SubG_{k}}\right)}^{G}=0$.*

### 3.2. Overall Framework of MGML

The framework of *MGML* includes two major steps: (1) Mining Informative Subgraphs. Our novel subgraph-mining technique *Entropy-based Subgraph Mining* will be utilized in this part and it includes the following steps. Firstly, *gSpan* will be utilized to generate all subgraphs in the dataset. Secondly, entropies of all subgraphs will be calculated and ranked according to our informative-subgraph criterion based on information entropy. We will discuss all details in Section 3.3; (2) Building Classifier. Top-ranked subgraphs will be used as classifying features. Graphs can be represented as instances based on what kinds of classifying features (a.k.a. informative subgraphs) that they contain, so graphs can be represented as multiple instances. Thus, *Multi-Graph Multi-Label* degenerates to *Multi-Instance Multi-Label*. After that, we will utilize *MIML-ELM*, an efficient and effective *MIML* algorithm to build a classifier. We will discuss all details in Section 3.4.

### 3.3. Mining Informative Subgraphs

In this section, we will discuss the evaluation of informative subgraphs and the algorithm how to mine them.

#### 3.3.1. Evaluation of Informative Subgraphs

Let us reconsider the example in Figure 3. Although another subgraph B−C has the frequency of only three, it appears three times in four positive graphs and does not appear in the negative ones at all, so it can stand for the trait of the positive class and is suitable to be regarded as a classifying feature. Generally, if a subgraph appears frequently in one of the classes but hardly appears in the other class, according to the definition of information entropy, this subgraph has low entropy. Thus, the subgraphs that have low entropy are the informative subgraphs that we need. We will give the formal definition of the informative subgraph in the following. Firstly, we will define it in the single-label problem for the ease of understanding and then expand it to a multi-label problem.

Firstly, we give the definition of information entropy for subgraph in the single-label problem. Assume that there is a set of graphs $Set_{G}=\left\{G_{1},\cdots ,G_{m}\right\}$. Each graph $G_{i}\left(i\in \left[1,m\right]\right)$ is only marked with a single label and the label is either positive or negative. $Set_{SubG}$ is the complete set of subgraphs mined from $Set_{G}$, which is denoted as $Set_{SubG}=\left\{SubG_{1},\cdots ,SubG_{n}\right\}$. For each subgraph $SubG_{j}\left(j\in \left[1,n\right]\right)$, the set of super-graphs for it is $Set_{G}^{j}=\left\{G_{1}^{j},\cdots ,G_{u}^{j}\right\}$. ${\#}_{pos}$ is the number of positive graphs in $Set_{G}^{j}$ and ${\#}_{neg}$ is the number of negative graphs in $Set_{G}^{j}$ (${\#}_{pos}+{\#}_{neg}=u$). Since each graph $G_{k}^{j}\left(k\in \left[1,u\right]\right)$ in $Set_{G}^{j}$ is the super-graph of $SubG_{j}$, the possibility of $SubG_{j}$ appearing in a positive (or negative) class equals the percentage of positive (or negative) graphs in $Set_{G}^{j}$ (based on Definition 2). Thus, the information entropy of subgraph $SubG_{j}$ is $E_{j}=-p_{pos}\log_{2}\left(p_{pos}\right)-p_{neg}\log_{2}\left(p_{neg}\right)$. $p_{pos}$ is the possibility of $SubG_{j}$ appearing in a positive class and $p_{pos}=\frac{{\#}_{pos}}{u}$; $p_{neg}$ is the possibility of $SubG_{j}$ appearing in a negative class and $p_{neg}=\frac{{\#}_{neg}}{u}$. The information entropy for the set of subgraphs $Set_{SubG}$ is $Set_{E(SubG)}=\left\{E_{1},\cdots ,E_{n}\right\}$.

Furthermore, the following is the definition of information entropy for a subgraph in a multi-label problem. Assume that there is a set of graphs $Set_{G}=\left\{G_{1},\cdots ,G_{m}\right\}$ and each graph $G_{i}\;\left(i\in \left[1,m\right]\right)$ is marked with a set of labels $Set_{L_{i}}=\left\{L_{i,1},\cdots ,L_{i,t}\right\}$. $Set_{SubG}$ is the complete set of subgraphs mined from $Set_{G}$, which is denoted as $Set_{SubG}=\left\{SubG_{1},\cdots ,SubG_{n}\right\}$. For each subgraph $SubG_{j}\;\left(j\in \left[1,n\right]\right)$, it has a set of information entropy $Set_{E_{j}}=\left\{E_{j,1},\cdots ,E_{j,t}\right\}$ (*t* different kinds of information entropy for *t* different labels). We define that, for each subgraph, the information entropy in a multi-label problem is the average entropy of all labels. Thus, for the subgraph $SubG_{j}$, the information entropy in a multi-label problem is $E_{j}=avg\left\{E_{j,1},\cdots ,E_{j,t}\right\}$. In the case of multi-label, the information entropy for the set of subgraphs $Set_{SubG}$ is denoted as $Set_{E(SubG)}=\left\{E_{1},\cdots ,E_{n}\right\}$.

Lastly, $Set_{E(SubG)}$ will be ranked increasingly. The top-ranked subgraphs (i.e., the ones with lower entropy) are the informative subgraphs.

#### 3.3.2. Entropy-Based Subgraph Mining

Current algorithms about classifying graph-structure data can be categorized into two groups: one is based on global distance, including *graph kernel* [25,26], *graph embedding* [27] and *graph transformation* [28], which calculates the similarity rate of two graphs; the other one is based on subgraph feature [29], including *AGM*, *Gaston*, *gSpan* and *gHSIC*, which converts a set of subgraphs to feature vectors. It has been proved that the latter one is better. It converts subgraphs to vectors so that most of the current algorithms can be utilized in the graph-structure classification problem.

To mine informative subgraphs as classifying features, one of the straightforward approaches is to mine the complete set of subgraphs for the graph set and rank these subgraphs with the evaluation function in Section 3.3.1, but this approach will cause a problem: the number of subgraphs grows exponentially when the size of graph set grows, so the enumeration will be tough work. Alternatively, we use a *Depth-First-Search* (*DFS*) based on algorithm *gSpan* to generate all subgraphs, using our evaluation during the process.

The key idea of *gSpan* is to build a lexicographic order among graphs that need to be mined, and then give each graph a unique label named *minimum DFS (Depth-First-Search) code*. *gSpan* uses the strategy of depth-first search to mine the frequent subgraph with the *DFS* code. Each time it needs to generate a new subgraph, it just recurs the character string (i.e., *DFS* code), so a subgraph-mining problem can be transformed into a substring-matching problem. Thus, the *gSpan* performs better than previous similar algorithms.

Generally, *gSpan* is utilized for mining the frequent subgraphs, but we do not care about the frequency of subgraph. We only use the *gSpan* to traverse all subgraphs and evaluate the information entropy during the traversal. This is the general idea of *Entropy-based Subgraph Mining* (*ESM*). The detailed algorithm of *ESM* is described in Algorithms 1 and 2.

**Algorithm 1:** GraphSet_Projection

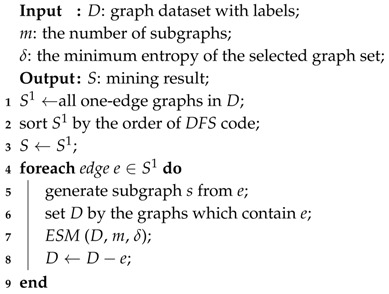



Note that Algorithm 1 invokes Algorithm 2 and Algorithm 2 is a recursion function. Firstly, it generates all subgraphs by traversing the graph from one edge (lines 4–9 in Algorithm 1). The search space is shrunk at the end of each turn (line 8 in Algorithm 1). Algorithm 2 traverses all graphs and generates all their subgraphs. It will stop when the code of the subgraph is not a minimum code (line 1 in Algorithm 2). The information entropy of the subgraph is computed and the result set is built by conducting Lines 4–11 in Algorithm 2. Lines 15–19 in Algorithm 2 grows the subgraph and does this function recursively.

### 3.4. Building Classifier

Since current algorithms of classifiers cannot be utilized directly in the graph-structure, after mining the informative subgraphs, we need to degenerate *Multi-Graph Multi-Label* (*MGML*) to *Multi-Instance Multi-Label* (*MIML*) based on Definition 3. The general idea is that, assuming there is a set of graphs $Set_{G}=\left\{G_{1},\cdots ,G_{m}\right\}$ and a set of informative subgraphs $Set_{InfoSubG}=\left\{InfoSubG_{1},\cdots ,InfoSubG_{n}\right\}$ mined from $Set_{G}$. For each graph $G_{i}\;\left(i\in \left[1,m\right]\right)$, it equals a feature vector $V_{i}=\left(x_{1},\cdots ,x_{n}\right)$ (a.k.a. instance) and the dimension of it is *n* (that equals to the number of informative subgraphs). For each informative subgraph $InfoSubG_{j}\;\left(j\in \left[1,n\right]\right)$, if $G_{i}$ is the super-graph of $InfoSubG_{j}$, $x_{j}$ in $V_{i}$ has $x_{j}=1$; otherwise, $x_{j}=0$. The labeled *MIML* set is $D=\{\left(B_{i},Y_{i}\right)|i=1,\cdots ,N\}$, where *N* is the number of bags in dataset *D* and $B_{i}=\left\{X_{1}^{i},\cdots ,X_{j}^{i},\cdots ,X_{n_{i}}^{i}\right\}$ is an instance bag with $n_{i}$ instances, $Y_{i}\in {\left\{0,1\right\}}^{M}$ is the label vector of bag $B_{i}$. Now, the *MGML* problem is degenerated to the *MIML* problem.


**Algorithm 2:**
*ESM*


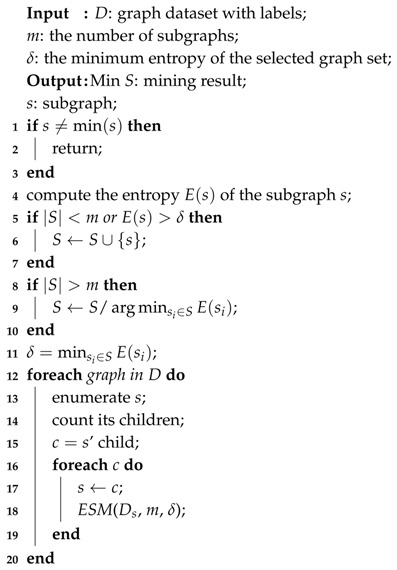



Traditionally, *Support Vector Machine* (*SVM*) is utilized to solve the *MIML*, but it has some drawbacks. First, *SVM* requires the user to input a great number of parameters. Second, using *SVM* to build a classifier may cause a high computing cost and the performance depends on the specific parameters that the user defined. Thus, we choose to use the *Extreme Learning Machine* (*ELM*) to solve the *MIML* problem. Firstly, *ELM* develops a theoretical guarantee to determine the number of clusters by *AIC* [30]. Secondly, a k-medoids cluster process is performed to transform from *Multi-Instance Multi-Label* into *Single-Instance Multi-Label*. Then, the Hausdorff distance [31] is used to measure the similarity between two different multi-instance bags. Based on the Hausdorff distance, *k*-medoids cluster divides the dataset into *k* parts, the medoids of which are $M_{1},\cdots ,M_{k}$. At last, we train the classifier for each label with *k*-dimensional numerical vectors.

### 3.5. Example of MGML

**Figure 4:**
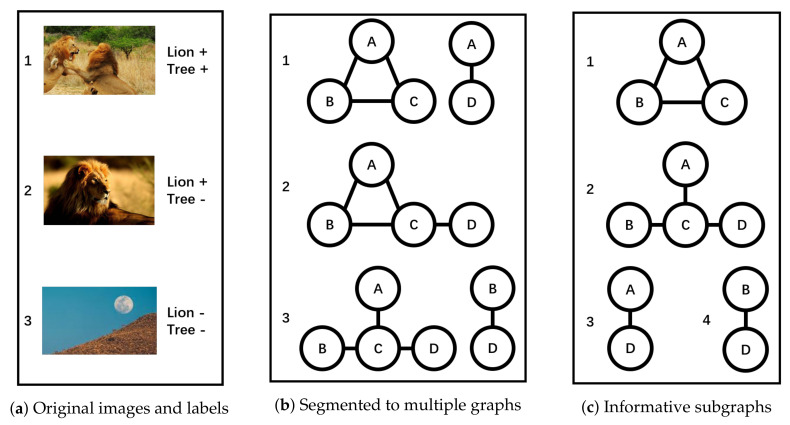
An example of MGML (1). (**a**) Original images and labels; (**b**) Segmented to multiple graphs; (**c**) Informative subgraphs.

In this section, we will give an explanatory example of *MGML*. In Figure 4a, there are three images and two labels. In image 1, label *Lion* is positive (+) and label *Tree* is positive (+). In image 2, label *Lion* is positive (+) and label *Tree* is negative (−). In image 3, label *Lion* is negative (−) and label *Tree* is negative (−). The following are the brief steps to build a classifier with *MGML*.

**Figure 5:**
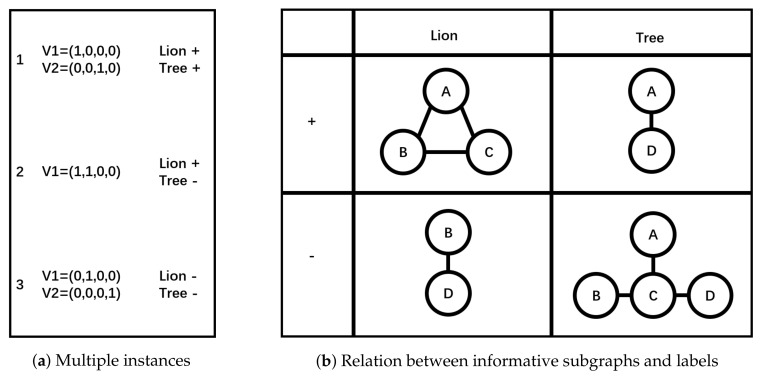
An example of MGML (2). (**a**) Multiple instances; (**b**) Relation between informative subgraphs and labels.

Firstly, segment these images into multiple graphs like Figure 4b. Secondly, utilize *Entropy-based Subgraph Mining* to mine informative subgraphs. The result is in Figure 4c. Thirdly, transform *Multi-Graph* to *Multi-Instance*. The result is in Figure 5a. Lastly, utilize *MIML-ELM* to build a classifier. The formula (i.e., relation) between informative subgraphs and labels is in Figure 5b. For example, subgraph A−D is the trait when label *Tree* is negative (−).

**Figure 6:**
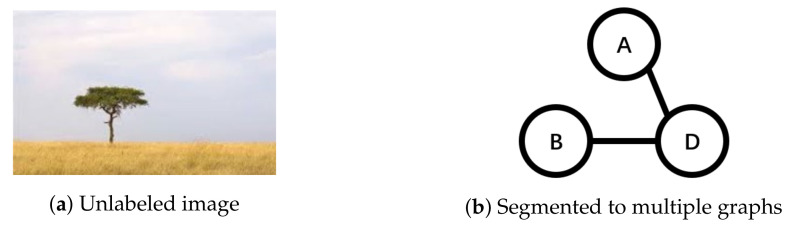
An example of MGML (3). (**a**) Unlabeled image; (**b**) Segmented to multiple graphs.

Assume that there is a new image without labels in Figure 6a. Segment the image into multiple graphs like Figure 6b. If we want to add labels for it with our *MGML* classifier, we just need to see what kinds of classifying features (i.e., the informative subgraphs in the previous step) it contains. It contains A−D and B−D, so it should have a negative (−) label *Lion* and a positive (+) label *Tree*.

## 4. Experimental Section

The following experiments are performed on a PC running Linux with an Intel dual-core CPU (2.60 GHz) (Shenyang, China) and 16 GB memory.

### 4.1. Datasets

Experiments are performed on three image datasets. These datasets have different sizes, including a small size of dataset named *MSRC v2 (Microsoft Research Cambridge)* [32], a middle size of dataset named *Scenes* [22,33] and a large size of dataset named *Corel5K* [34].

The summary of three datasets is given in Table 1.

We use two ways to segment the original datasets: the first one is to segment each image into six graph structures and each graph has eight nodes and 12 edges roughly (6×8×12); the second one is to segment each image into six graph structures and each graph has nine nodes and 15 edges roughly (6×9×15). In short, graphs in 6×9×15 set are more complex to mining than the 6×8×12 one.

In the following experiments, each dataset will be randomly divided to a training dataset and a testing dataset and the ratio of them is about 2:1. The training dataset will be used to build the classifier and the testing dataset will be used to evaluate its performance. All experiments repeatedly run thirty times, and each time the training dataset and the testing dataset will be divided randomly.

### 4.2. Evaluation Criteria

Assume that there is a test dataset $S=\{\left(X_{1},Y_{1}\right),\left(X_{2},Y_{2}\right),\cdots ,\left(X_{p},Y_{p}\right)\}$. $h(X_{i})$ denotes a set of correct labels of $X_{i}$; $h(X_{i},y)$ denotes the confidence for *y* to be a correct label of $X_{i}$; $rank^{h}\left(X_{i},y\right)$ denotes the rank of *y* derived from $h(X_{i},y)$. The following are four evaluation criteria to measure the performance of our *MGML* algorithm.

1. RankingLoss $=\frac{1}{p}\sum_{i=1}^{p}\frac{1}{\left|\right|Y_{i}\left|||\right|\bar{Y_{i}}\left|\right|}\left|\left\{\left(y_{1},y_{2}\right)|h\left(X_{i},y_{1}\right)\leq h\left(X_{i},y_{2}\right),\left(y_{1},y_{2}\right)\in Y_{i}\times \bar{Y_{i}}\right\}\right|$, where $Y_{i}$ denotes the complementary set of $Y_{i}$ in *Y*. It indicates the average fraction of labels that are misordered for a specific object. The smaller the value of *RankingLoss* is, the better the performance reaches. When it equals to 0, the performance reaches perfect.
2. OneError $=\frac{1}{p}\sum_{i=1}^{p}\left[\arg\max_{y\in Y}h\left(X_{i},y\right)\notin Y_{i}\right]$. It indicates the average time that the top labels in the rank are not the correct ones for a specific object. The smaller the value of *OneError* is, the better the performance reached. When it equals 0, the performance can reach perfection.
3. Coverage $=\frac{1}{p}\max_{y\in Y}rank^{h}\left(X_{i},y\right)-1$. It indicates the average number of labels in the rank that need to be included to cover all the correct labels for a specific object. The smaller the value of *Coverage* is, the better the performance.
4. Average Precision $avgprec_{S}\left(h\right)=\frac{1}{p}\sum_{i=1}^{p}\frac{1}{|Y_{i}|}\sum_{y\in Y_{i}}\frac{\left|\{y^{\prime }|rank^{h}\left(X_{i},\;y^{\prime }\right)\;\leq \;rank^{h}\left(X_{i},\;y^{\prime }\right)\;\leq \;rank^{h}\left(X_{i},\;y\right),\;y^{\prime }\in Y_{i}\}\right|}{rank^{h}\left(X_{i},\;y\right)}$. It indicates the the average fraction of correct labels in all labels $Y_{i}$. The larger the value of *Average Precision* is, the better the performance. When it equals 1, the performance can reach perfection.

### 4.3. Effectiveness

In this section, we will use ①, ② and ③ to mark different algorithms for the differentiation, and we will call our *MGML* algorithm ① *MGML-ELM*, which means that “the *MGML* algorithm using *MIML-ELM*”.

Currently, there are no other methods of *MGML* learning that can be compared to our algorithm. Thus, we use the ③ *MIML-SVM*, one of the state-of-the-art algorithms for *MIML* learning, as the competitor. In addition, we use the ② *MGML-SVM* as the baseline algorithm for competitions. The ② *MGML-SVM* algorithm is generally the same as the ① *MGML-ELM*. It also needs to degenerate the *MGML* problem into the *MIML* problem, but then it uses the *SVM* instead of *ELM* in the next step.

The parameter of ① *MGML-ELM* is the number of hidden layer (*hn*), which is respectively set to 50, 100, 150, 200; the parameter of ② *MGML-SVM* and ③ *MIML-SVM* is the penalty factor of *Gaussian* kernel (*Cost*), which is respectively set to 1, 2, 3, 4, 5. The final results on average are in Table 2, Table 3 and Table 4. The bold one means the best performance for every criterion. The ↓ indicates *the smaller the better*, while the ↑ indicates *the larger the better*.

For ease of reading, we use ①, ② and ③ in this paragraph to respectively indicate ① *MGML-ELM* ② *MGML-SVM* and ③ *MIML-SVM*. As seen from the results in Table 2, in the dataset *MSRC v2*, our algorithm ① performs best when the hn=100 and the precision reaches 82%, *RankingLoss* reaches 0.07, *OneError* reaches 0.17 and *Coverage* reaches 3.93, while the precision of ② reaches 76% at best when Cost=5, *RankingLoss* reaches 0.14, *OneError* reaches 0.19 and *Coverage* reaches 6.19, and ③ reaches 75% at best when Cost=5, *RankingLoss* reaches 0.10, *OneError* reaches 0.24 and *Coverage* reaches 4.88.

As seen from the results in Table 3, in the dataset *Scenes*, our ① reaches 81% at best when the hn=150, *RankingLoss* reaches 0.17, *OneError* reaches 0.30 and *Coverage* reaches 1.78, while ② reaches 74% at best when Cost=3, *RankingLoss* reaches 0.29, *OneError* reaches 0.34 and *Coverage* reaches 1.28, and ③ reaches 25% at best when Cost=5, *RankingLoss* reaches 0.92, *OneError* reaches 0.96 and *Coverage* reaches 3.87.

As seen from the results in Table 4, in the dataset *Corel5K*, our ① reaches 23% at best when the hn=200, *RankingLoss* reaches 0.21, *OneError* reaches 0.75 and *Coverage* reaches 119.31, while ② reaches 13% at best when Cost=5, *RankingLoss* reaches 0.31, *OneError* reaches 0.88 and *Coverage* reaches 144.87, and ③ reaches 22% at best when Cost=5, *RankingLoss* reaches 0.19, *OneError* reaches 0.77 and *Coverage* reaches 110.52. Thus, we can say that our ① *MGML-ELM* achieves the best performance in all cases.

### 4.4. Efficiency

In this section, we test the efficiency of our *MGML* algorithm. For either *MGML* or *MIML*, as long as the input numbers of features for *ELM* and *SVM* are the same, the runtime for both of these two algorithms will be equal. Therefore, the only way to test the efficiency is focusing on the part of mining the classifying features. Our *MGML* uses an innovate technique *Entropy-based Subgraph Mining* (*ESM*) to mine the informative subgraphs as the features, while the *gMGFL* of Jia Wu mines the frequent subgraphs as the features, which is based on the boosting *gSpan* named *gboost* [35]. We respectively compared the time of our *ESM* for mining subgraphs with *gboost* in three datasets: *MSRC v2*, *Scenes* and *Corel5K*.

**Figure 7:**
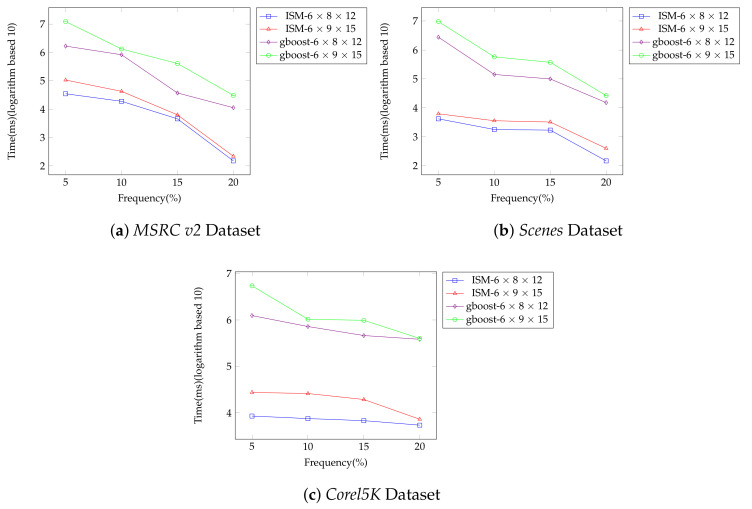
Results of efficiency experiments. (**a**) *MSRC v2* Dataset; (**b**) *Scenes* Dataset; (**c**) *Corel5K* Dataset.

We implement *ESM* and *gboost* in two kinds of segmented datasets (6×9×15 and 6×8×12) and the minimum frequency is set to 5%, 10%, 15%, and 20%. In *ESM*, if the frequency of a graph is lower than the minimum frequency we set, we will not calculate the entropy for this graph; while, in *gboost*, if the frequency of a graph is lower than the minimum frequency, we will not continue to mine the subgraphs for this graph. In short, the lower the minimum frequency is, the more graphs the algorithm needs to mine. The final results in average are in Figure 7a–c. The time results are logarithmic.

As seen from the results in Figure 7a–c, *gboost* takes hours to mine the huge datasets, but *ESM* takes only several minutes to generate results. For example, in the dataset *MSRC v2* (6×9×15), when the minimum frequency is set to 5%, *ESM* takes two minutes to mine the results while *gboost* takes 4 h to do that; in the dataset *Scenes* (6×9×15), when the minimum frequency is set to 10%, *ESM* takes 4 s to mine the results while *gboost* takes 10 min to do that; in the dataset *Corel5K* (6×9×15), when the minimum frequency is set to 15%, *ESM* takes 19 s to mine the results while *gboost* takes 16 min to do that. These figures show that *ESM* achieves better performance by 100–1000 times in comparison with *gboost*.

## 5. Conclusions

In this paper, we have shown how the *Multi-Graph Multi-Label Learning* (*MGML*) works. The *MGML* uses the more precise structures, multiple graphs, instead of instances to represent an image, which can improve the accuracy of classification dramatically in the latter step. In addition, it uses multiple labels as the output to eliminate the ambiguity of description. Furthermore, we use our technique *Entropy-based Subgraph Mining* to mine the informative subgraphs, rather than simply regard frequent subgraphs as informative subgraphs. Then, we show how to degenerate *MGML* to *MIML*. At last, we use the *MIML-ELM* as the base classifier. Extensive experimental results prove that *MGML* achieves a good performance in three image datasets with different sizes. What we are interested in for the future steps is to improve the performance in the dataset that has sparse labels by using other algorithms as the classifier.

## Figures and Tables

**Table 1:** The summary of datasets.

Dataset	# of Bags	# of Labels	Labels Per Bag
*MSRC v2*	591	23	2.5
*Scenes*	2000	5	1.2
*Corel5K*	5000	260	3.5

**Table 2:** *MSRC v2* Dataset.

*MSRC v2*		Evaluation Criterion			
		*RankingLoss*↓	*OneError*↓	*Coverage*↓	*Average Precision*↑
① *MGML-ELM*	hn = 50	0.070079	0.183039	3.92824	0.809013
	**hn = 100**	**0.071367**	**0.172589**	**3.928934**	**0.820388**
	hn = 150	0.075182	0.19797	3.989848	0.804771
	hn = 200	0.07181	0.187817	3.86802	0.808192
② *MGML-SVM*	Cost = 1	0.154664	0.19797	7.35533	0.754622
	Cost = 2	0.171189	0.229066	7.122844	0.761665
	Cost = 3	0.183357	0.233503	7.634518	0.735131
	Cost = 4	0.140284	0.219557	7.628352	0.735253
	**Cost = 5**	**0.137361**	**0.187817**	**6.187817**	**0.762115**
③ *MIML-SVM*	Cost = 1	0.105581	0.295073	5.267476	0.710714
	Cost = 2	0.104209	0.292204	5.218223	0.715079
	Cost = 3	0.100998	0.253995	5.044584	0.721987
	Cost = 4	0.097587	0.247775	4.890471	0.73787
	**Cost = 5**	**0.0955**	**0.240682**	**4.880897**	**0.745322**

**Table 3:** *Scenes* Dataset.

*Scene*		Evaluation Criterion			
		*RankingLoss*↓	*OneError*↓	*Coverage*↓	*Average Precision*↑
① *MGML-ELM*	hn = 50	0.16927	0.318	1.771	0.798919
	hn = 100	0.172833	0.33	1.81	0.798367
	**hn = 150**	**0.165**	**0.304**	**1.78**	**0.811867**
	hn = 200	0.160667	0.312	1.762	0.8102
② *MGML-SVM*	Cost = 1	0.299667	0.806	1.324	0.555683
	Cost = 2	0.298835	0.434	1.324	0.694689
	**Cost = 3**	**0.2935**	**0.34**	**1.284**	**0.7401**
	Cost = 4	0.252933	0.36	1.312	0.630968
	Cost = 5	0.237167	0.458	1.062	0.71515
③ *MIML-SVM*	Cost = 1	0.910205	0.950815	3.810687	0.242251
	Cost = 2	0.91073	0.95178	3.844675	0.242479
	Cost = 3	0.91348	0.956172	3.864988	0.245989
	Cost = 4	0.914378	0.957471	3.86676	0.246726
	**Cost = 5**	**0.917939**	**0.958322**	**3.867907**	**0.249013**

**Table 4:** *Corel5K* Dataset.

*Corel5K*		Evaluation Criterion			
		*RankingLoss*↓	*OneError*↓	*Coverage*↓	*Average Precision*↑
① *MGML-ELM*	hn = 50	0.202493	0.750168	113.8968	0.224968
	hn = 100	0.197103	0.743487	113.354709	0.224146
	hn = 150	0.21584	0.755511	120.549098	0.219783
	**hn = 200**	**0.205424**	**0.751503**	**119.306613**	**0.225752**
② *MGML-SVM*	Cost = 1	0.30124	0.857229	139.0005	0.120073
	Cost = 2	0.301264	0.86724	140.9756	0.121792
	Cost = 3	0.301906	0.868129	141.8067	0.122804
	Cost = 4	0.304838	0.870358	143.3142	0.123633
	**Cost = 5**	**0.307872**	**0.880693**	**144.8687**	**0.128766**
③ *MIML-SVM*	Cost = 1	0.191867	0.768118	110.4207	0.217195
	Cost = 2	0.191899	0.768204	110.4322	0.217209
	Cost = 3	0.191922	0.768299	110.4657	0.217219
	Cost = 4	0.191978	0.768416	110.4719	0.217285
	**Cost = 5**	**0.191997**	**0.768899**	**110.5187**	**0.217299**
